# Hygienic disposal of stools and risk of diarrheal episodes among children aged under two years: Evidence from the Ghana Demographic Health Survey, 2003–2014

**DOI:** 10.1371/journal.pone.0266681

**Published:** 2022-04-07

**Authors:** John Tetteh, Isaac Adomako, Emilia Asuquo Udofia, Elom Yarney, Henry Quansah, Anita Ohenewa Yawson, Akye Essuman, Alfred Edwin Yawson

**Affiliations:** 1 Department of Community Health, University of Ghana Medical School, College of Health Sciences, University of Ghana, Accra, Ghana; 2 Ground Floor Surgical Intensive Care Unit, Department of Anaesthesia, Korle-Bu Teaching Hospital, Accra, Ghana; 3 School of Nursing and Midwifery, College of Health Sciences, University of Ghana, Accra, Ghana; Xiamen University - Malaysia Campus: Xiamen University - Malaysia, MALAYSIA

## Abstract

**Background:**

Most childhood diarrheal illnesses are a result of the faeco-oral transmission of infected food, water, and unclean fingers. The present paper was conducted to estimate the prevalence of hygienic disposal of stools (HDS) and its associated factors, and further quantify the impact of HDS on diarrheal diseases among children under two years.

**Methods:**

A cross-sectional design was used to evaluate three rounds of the Ghana Demographic Health Survey (GDHS) from 2003–2014 involving 4869 women with children aged under two years. The outcomes were prevalence of HDS and diarrheal diseases. Poisson regression model was employed to assess risk factors associated with HDS and dominance analysis was used to rank the important risk factors. Inverse Probability Weighting Poisson Regression Adjustment (IPWPRA) with Propensity Score 1:1 density kernel-based matching was employed to assess impact.

**Results:**

The pooled prevalence rate of HDS was 26.5%(95%CI = 24.6–28.4) and it ranged from 18.7% (95%CI = 16.4–21.2) in 2014 to 38.8%(95%CI = 35.3–42.4) in 2003. Diarrhea diseases pooled prevalence was 17.9%(95%CI = 16.4–19.5) and ranged from 13.3%(95%CI = 11.1–15.9) in 2014 to 25.4%(95%CI = 22.2–28.9) in 2003. The overall growth rate for HDS and prevalence of diarrhea diseases, decreased by 21.6% and 11.4% respectively. The most important risk factors of HDS from dominance analysis included; age of the child, wealth index, and differences in region. From pooled data wealth index, increasing age of the child, and regional disparity constituted approximately 72% of the overall impact (Weighted Standardized Dominance Statistics (WSDS) = 0.30, 0.24, and 0.19 respectively). In 2014, they constituted approximately 79% (WSDS = 0.139, 0.177 and 0.471 respectively). The average prevalence of diarrheal diseases among children of women who practiced HDS reduced over the period of the GDHS compared to those whose mothers did not practice HDS [2008 ATE(95%CI) = -0.09(-0.16–0.02), 2014 ATE(95%CI) = -0.05(-0.09–0.01) and Pooled data ATE(95%CI) = -0.05(-0.09–0.02)].

**Conclusion:**

This analysis has provided empirical evidence of the impact of practicing HDS in Ghana from a national household survey. Implementation of the WASH agenda in this low-income setting requires a synergy of interventions and collaborations of actors (government, private and development partners) to improve water and sanitation facilities and to increase hygiene education to prevent the spread of diseases including diarrhea by 2025.

## Introduction

Adequate and equitable access to improved disposal of liquid waste to end open defecation remains a cross-cutting problem in developing countries [1]. The World Health Organization (WHO) report demonstrates that currently, basic sanitary services such as toilets and latrines are unavailable to two billion population [1]. Children’s stools are given less attention in Sub-Saharan Africa and many other impoverished nations because they are deemed ‘harmless’ relative to adults’ stool while having a larger pathogenic load [2, 3]. Sanitation seeks to prevent contamination of the environment by excreta and curb the spread of diarrhea-causing pathogens, however, improved sanitation coverage in Ghana is less than 15% [3, 4]. While the country has made remarkable progress in achieving access to improved water supply, the same cannot be said for sanitation [5].

Many sanitation interventions globally overlooked children’s stool disposal in many countries [2]. In Ghana, reducing open defecation and improving child excrement disposal practices require priority attention [6], as unsafe disposal of young children’s stools makes them susceptible to diarrheal diseases due to their oral behaviour [2, 7–10]. However, the sanitation literature has placed a greater focus on the availability of toilets and less on user behavior, such as the proper disposal of child excrement [8]. Available literature suggests that access to improved sanitation does not necessarily lead to improved disposal of stool, especially in children [11, 12]. Unsafe practices such as stools left at the site of deposition, children observed to be handling stool, and washing of diapers in water bodies (such as rivers and streams), expose children to pathogens. This risk is due to their propensity to put their fingers in the mouth, come into contact with soil while playing or eating soil or food and substances contaminated with faeces [8].

Children’s, as well as adult’s stools, must be disposed off safely, due to its association with diarrhea diseases, especially in poor urban communities [13]. Diarrheal diseases are among the top five common causes of death in children, accounting for over 525000 deaths among children [14]. Most childhood diarrheal illnesses are as a result of the faeco-oral transmission of infected food, water, and unclean fingers [15, 16]. Seidu estimated the prevalence and assessed the factors associated with the safe disposal of children’s stools in Ghana [17]. Seidu’s study, however, did not address the impact of safe disposal of young children’s stool in the long term. This analysis estimates the prevalence of hygienic disposal of stools (HDS), explores associated factors, and further assesses the impact of practicing HDS on the risk of diarrhea diseases among children less than two years old using nationally representative data from the Ghana Demographic and Health Surveys (GDHS).

## Methods

### Study design and description

This analysis used cross-sectional study data from the Ghana Demographic and Health Survey (GDHS), which was conducted across the country in the then ten administrative regions. The GDHS is a nationally representative household surveys that offer data for a variety of population, health, and nutrition monitoring and impact evaluation variables. Data used for this analysis was from the fourth to sixth rounds of the GDHS. The major goals of the GDHS were to collect data on fertility and family planning behaviour, infant and child mortality, breastfeeding, antenatal care, children’s immunizations, and childhood diseases, nutritional status of mothers and children, use of maternal and child health services, and awareness and behaviour regarding AIDS and other STIs.

The fourth round of GDHS was conducted in 2003, and approximately 6,600 households were selected nationwide. The fifth round GDHS (2008) selected 12,000 households while the sixth round (2014) selected 12,810 households across the country. Both 2003 and 2008 used 412 Enumeration Areas (EAs) selected from the 2000 Ghana Population and Housing Census (GPHC) used as a frame for the sample whiles 2014 used 427 EAs selected from the 2010 GPHC. The frame was first stratified into the 10 administrative regions in the country, then into rural and urban EAs. All the study rounds adopted a two-stage stratified cluster sampling method to obtain the sample for each survey year. In the first stage, EAs were selected with probability proportional to the EA size and with independent selection in each sampling stratum. The second stage entailed taking systematic sampling from a list of households in each of the EAs that had been selected.

### Primary outcome

The study considered diarrhea disease as the primary outcome which GDHS measured subjectively. Participants living with a child under five years were asked whether the child had diarrhea diseases during the past two weeks preceding the survey. Answers included “Yes”, “No” and “Don’t know”. In this analysis, children aged two years or more and ‘Don’t know responses were excluded. The denominator has changed over time from children under age 5 to children under two in recent times [18].

### Secondary outcome

HDS was the secondary outcome considered in this study and was generated in two steps. First, we generated safe disposal of child’s stool from GDHS data. GDHS asked women with children under age two years the manner of disposal of the child’s last stool ‘Used toilet/latrine’, ‘pot/rinsed in toilet/latrine’, pot/rinsed into drain or ditch’, ‘throw into garbage’, ‘buried’, ‘rinsed away’, ‘use disposable diapers’, ‘use washable diapers’, ‘left in the open/not disposed off’, and ‘other’ were options provided. By GDHS definition, disposal is safe if the child used the toilet or latrine, stools are rinsed into the toilet or latrine, or stools are buried. Secondary, improved toilet facility was also generated and based on the definition by the WHO/UNICEF Joint Monitoring Programme (JMP) for Water Supply and Sanitation [18]. The toilet facility was classified as improved if not shared, flush to-piped sewer system, septic tank, pit latrine, and unspecified; pit latrine—ventilated improved pit, with slab and composting toilet. From the above two approaches, HDS was generated if a woman with a child(ren) under two years practiced safe disposal and had access to an improved toilet facility (coded as 1 and otherwise as 0).

### Data analysis

The first approach to data analysis was to denormalize the individual women sampling weight since authors merged GDHS data from 2003–2014. In the present analysis, women aged 15–49 years at the time of the survey were used to estimate the sampling fraction. Analysis adjusted for the nature of the design of the GDHS, thus, adjusting for the denormalized sampling weights, stratification, and the primary sampling unit.

Descriptive and test of independence analysis were performed by adopting the Rao-Scott test of independence to test the association of covariates with GDHS year of study among participants (S1 Table). Based on previous literature and additional variables in the dataset, 25 variables were identified a *priori*. Authors then employed Poisson regression method to assess the factors associated with HDS. Poisson regression was employed to estimate the adjusted prevalence (PR) ratio rather than odd ratio (OR). In a cross-sectional survey with prevalence rate 10% or more, the PR is the preferred choice for risk analysis [19].

After identifying significant factors associated with the secondary outcome variable from the Poisson regression, the authors employed a weighted dominance analysis (DA) to estimate the relative importance of significant factors associated with the secondary outcome variable. The Logit model in DA was employed which relies on estimating the coefficient of determination (R^2^) values of all possible combinations of explanatory variables (EVs) and measures the relative importance by adopting pairwise comparisons of all EVs in the model as they relate to the secondary outcome. DA is a statistical technique for comparing the relative importance of a predictor variable over another which is associated with an outcome variable [20].

Due to the cross-sectional design of the GDHS, a matching procedure was used to assess the impact of having access to hygienic disposal of stools on diarrhea disease. In order to study the effects of the exposure (hygienic disposal of stools), a matching procedure was used to select controls in a sample with the same covariate values as the treated sample [21]. The propensity score 1:1 number of matching per observation was adopted and the Epanechnikov kernel function was obtained to assess the impact. We then estimated the average treatment effect (ATE) of HDS on diarrhea disease using logit.

The conceptual framework defining the analytical process adopted for achieving the study objective can be found in S1 Fig. All analyses were performed using Stata 16.1 and a p-value <0.05 was deemed significant. The study relied on the “Strengthening of Epidemiological Observational Research Report” (STROBE) statement [22] in writing the manuscript as presented in S1 Table.

In addition, we estimated the overall growth rate of HDS and diarrheal diseases from 2003–2014 by adopting the formula $r=\left(\sqrt[{n}]{\frac{P_{1}}{P_{0}}}\right)-1$; where r = growth rate, P_1_ the current estimates in 2014, and P_0_ = the past estimate in 2003.

Therefore, HDS $r=\left(\sqrt[{3}]{\frac{18.7}{38.8}}\right)-1$; thus *SISD r* = -0.2159 and Diarrhea $r=\left(\sqrt[{3}]{\frac{13.3}{19.1}}\right)-1$; thus *Diarrhea r* = -0.1136.

The rate of percentage change within the periods was calculated by adopting the formula $c=\left(\frac{x_{2}-x_{1}}{x_{1}}\right)*100$; where c represents the relative change while x_2_ and x_1_ denote current and initial values respectively.

### Ethical considerations

The GDHS protocol was reviewed and approved by the Ghana Health Service Ethical Review Committee and the ICF Institutional Review Board examined. The ICF IRB guarantees that the survey follows all U.S. regulations. Regulations for the protection of human subjects issued by the Department of Health and Human Services (45 CFR 46). Individual women’s written consent was obtained during the data collection process for all participants. Privacy and confidentiality were strictly adhered to.

## Results

### Prevalence of hygienic disposal of stools and diarrheal diseases

Table 1 demonstrates that the pooled prevalence of HDS was 26.5(95%CI = 24.6–28.4) and it ranged from 18.7%(95%CI = 16.4–21.2) in 2014 to 38.8%(95%CI = 35.3–42.4) in 2003; while, the pooled diarrhea disease prevalence was 17.9%%(95%CI = 16.4–19.5) with a range from 13.3%(95%CI = 11.1–15.9) in 2014 to 25.4%(95%CI = 22.2–28.9) in 2003. The overall growth rate for HDS and diarrhea diseases was decreased by 21.6% and 11.4% respectively (Table 1).

**Table 1:** Prevalence and percentage change in access to hygienic disposal of stools and diarrhea diseases among women with children under two years in Ghana, GDHS 2003–2014.

Year	Hygienic disposal of stools	Diarrhea disease
	Prev[95%CI]	Prev[95%CI]
2003	38.8[35.3–42.4]	19.1[17.1–21.2]
2008	27.2[23.6–31.0]	25.4[22.3–28.9]
2014	18.7[16.4–21.2]	13.3[11.1–15.9]
Pooled	26.5[24.6–28.4]	17.9[16.4–19.5]
Growth Rate	(21.6)	(11.4)
**Percentage change**		
2003–2008	-11.6[-16.7–6.4]***	6.3[2.4–10.2]***
2003–2014	-20.1[-24.4–15.7]***	-5.7[-8.9–2.6]***
2008–2014	-8.5[12.9–4.0]***	-12.1[-16.1–8.0]***

Prev = prevalence estimate, 95%CI = 95% confidence limits about the estimate; P-value Notation

***p-value ≤ 0.001.

The change in HDS prevalence decreased significantly within 2003–2014 (changes from 2003–2008, 2003–2014, and 2008–2014 were 11.6%, 20.1%, 8.5% reduction rate respectively). The change in diarrhea prevalence increased significantly at a rate of 6.3%(95%CI = 2.4–10.2) from 2003–2008. However, the periods between 2003–2014 and 2008–2014 showed significant declines in prevalence (5.7% and 12.1% respectively) (Table 1). The projected figure for HDS and diarrhea infection for the next GDHS were 8.1% and 13.5% respectively (S2 Table).

### Child, individual, and household characteristics associated with access to hygienic disposal of stools among women with children under two years in Ghana, GDHS 2003–2014

Overall and consistently, characteristics including child’s age, region, wealth index, and electricity in the household (HH) were factors significantly associated with HDS. Increasing age of the child significantly increased the likelihood of HDS [Pooled adjusted Prevalence Ratio (aPR) = 1.04, 95%CI = 1.03–1.05]. The regional disparity from pooled data showed that Western, Central, Greater Accra, Volta, Eastern, Ashanti, and Brong Ahafo significantly increased the likelihood of HDS compared with their counterparts who reside in the Northern region (Table 2). In addition, as the wealth index increases, the adjusted HDS prevalence ratio increases significantly. Generally, participants with electricity in the HH had a significantly higher chance of disposing of children’s stools safely and used improved latrines [aPR(95%CI) = 1.53(1.30–1.79)] (Table 2).

**Table 2:** Child, individual and household characteristics associated with access to hygienic disposal of stools among women with children under two years in Ghana, GDHS 2003–2014.

Variable	GDHS year of study			
	2003	2008	2014	Pooled
	aPR[95%CI]	aPR[95%CI]	aPR[95%CI]	aPR[95%CI]
**Child Characteristics**				
**Sex of child**				
Male	1	1	1	1
Female	1.08[1.05–1.08]	1.10[0.92–1.32]	1.08[0.84–1.39]	1.09[1.00–1.49]*
**Age of child**	1.06[1.05–1.08]***	1.04[1.02–1.06]***	1.03[1.01–1.04]**	1.04[1.03–1.05]***
**Birth order**				
1st	1	1	1	1
2nd	0.91[0.74–1.11]	1.07[0.79–1.46]	1.54[1.08–2.20]**	1.10[0.92–1.30]
3rd	0.94[0.74–1.19]	1.27[0.85–1.89]	1.34[0.85–2.11]	1.03[0.84–1.27]
4th+	0.98[0.71–1.35]	1.59[0.94–2.68]	1.88[1.03–3.41]*	1.27[0.96–1.67]
**Multiple birth**				
No	1	1	1	1
Yes	1.23[0.77–1.99]	1.34[0.55–3.26]	1.31[0.82–2.11]	1.35[1.00–1.83]
**Women characteristics**				
**Age of mother**	1.01[0.98–1.02]	1.00[0.97–1.03]	1.01[0.98–1.05]	1.00[0.99–1.02]
**Educational level**				
Higher	1	1	1	1
No education	0.71[0.37–1.38]	0.49[0.27–0.88]**	0.83[0.43–1.62]	0.87[0.59–1.27]
Primary	0.88[0.47–1.67]	0.47[0.27–0.82]**	0.93[0.50–1.73]	0.97[0.68–1.38]
Secondary	0.91[0.50–1.69]	0.78[0.49–1.26]	1.09[0.63–1.89]	1.10[0.79–1.53]
**Religion**				
Islam	1	1	1	1
Christian	2.43[1.00–5.93]*	1.12[0.76–1.64]	0.81[1.12–3.58]**	1.17[0.90–1.51]
No religion	0.78[0.25–2.37]	0.46[0.22–0.99]*	0.90[0.46–1.74]	0.61[0.41–0.90]*
**Relationship to HH head**				
Wife	1	1	1	1
Head	0.82[0.55–1.21]	0.98[0.53–1.80]	2.01[1.12–3.58]**	1.17[0.87–1.57]
Other	1.17[0.81–1.67]	0.90[0.51–1.57]	0.90[0.46–1.74]	0.98[0.73–1.31]
**Currently pregnant**				
No	1	1	1	1
Yes	0.82[0.59–1.14]	0.91[0.55–1.50]	1.44[0.93–2.21]	1.08[0.84–1.38]
**Number of living children**				
**Wanted last child**				
Wanted then	1	1	1	1
Wanted later	0.98[0.84–1.14]	1.00[0.80–1.27]	1.29[0.99–1.69]	1.09[0.96–1.23]
Wanted nomore	1.09[0.92–1.28]	0.72[0.53–0.97]*	1.08[0.72–1.61]	1.10[0.95–1.03]
**Currently breastfeeding**				
No	1	1	1	1
Yes	1.22[1.00–1.41]	1.07[0.78–1.46]	1.03[0.78–1.36]	1.19[1.02–1.39]*
**Current marital status**				
Married	1	1	1	1
Never married	1.03[0.75–1.42]	1.71[1.04–2.83]*	0.87[0.53–1.42]	0.98[0.75–1.29]
DSW	1.04[0.77–1.41]	1.46[0.89–2.39]	0.53[0.29–0.99]*	0.96[0.74–1.24]
**Currently working**				
Yes	1	1	1	1
No	0.96[0.79–1.16]	1.00[0.73–1.37]	1.38[1.11–1.73]***	1.05[0.91–1.21]
**Reads newspaper/magazine**				
Yes	1	1	1	1
No	0.92[0.76–1.12]	1.31[0.98–1.76]	1.20[0.85–1.69]	1.02[0.87–1.21]
**Listen to radio**				
Yes	1	1	1	1
No	0.89[0.69–1.13]	1.19[0.82–1.72]	0.97[0.70–1.34]	0.93[0.78–1.12]
**Watch television**				
Yes	1	1	1	1
No	1.00[0.87–1.15]	0.74[0.54–1.02]	1.22[0.75–1.99]	0.91[0.79–1.05]
**HH characteristics**				
**Sex of HH head**				
Female	1	1	1	1
Male	0.97[0.67–1.42]	1.06[0.63–1.77]	1.22[0.75–1.99]	1.04[0.80–1.35]
**Age of household**				
≤29	1.15[0.83–1.60]	0.82[0.48–1.39]	0.45[0.26–1.78]	0.70[0.54–1.90]
30–39	1.15[0.88–1.50]	0.82[0.53–1.28]	0.81[0.54–1.23]	0.84[0.68–1.03]
40–49	1.14[0.88–1.46]	1.01[0.69–1.49]	0.88[0.63–1.24]	0.97[0.81–1.17]
**Region**				
Northern	1	1	1	1
Western	3.34[1.94–5.76]***	2.28[0.98–5.31]	17.29[5.72–52.26]***	4.09[2.48–6.76]***
Central	3.47[1.97–6.11]***	2.27[0.95–5.44]	12.56[4.26–37.06]***	3.58[2.19–5.85]***
Greater Accra	1.97[1.07–3.62]*	1.89[0.80–4.47]	7.00[2.21–22.18]***	2.39[1.42–4.02]***
Volta	2.09[1.18–3.72]**	2.28[0.94–5.50]	8.82[2.91–26.70]***	2.49[1.51–4.14]***
Eastern	3.79[2.21–6.51]***	3.58[1.59–8.05]**	16.93[5.70–50.24]***	4.78[2.94–7.75]***
Ashanti	3.23[1.91–5.47]***	2.18[0.96–4.94]	6.67[2.15–20.68]***	2.94[1.79–4.83]***
Brong Ahafo	3.49[2.09–5.81]***	2.68[1.18–6.07]**	11.33[3.91–32.86]***	3.93[2.45–6.32]***
Upper East	4.50[2.60–7.81]***	1.06[0.42–2.67]	1.97[0.58–6.64]	0.64[0.33–1.24]
Upper West	0.49[0.25–0.99]*	0.86[0.31–2.39]	5.64[1.84–17.24]***	1.09[0.63–1.86]
**Place of residence**				
Urban	1	1	1	1
Rural	0.99[0.81–1.19]	0.85[0.62–1.14]	0.98[0.75–1.29]	1.01[0.86–1.19]
**Wealth index**				
Poorest	1	1	1	1
Poorer	1.43[1.11–1.84]**	2.17[1.09–4.34]*	1.62[0.94–2.80]	1.69[1.31–2.18]***
Middle	1.65[1.23–2.22]***	3.73[1.79–7.78]***	1.63[0.87–3.04]	2.35[1.77–3.12]***
Richer	1.99[1.45–2.73]***	5.71[2.66–12.26]***	1.67[0.88–3.20]	3.16[2.34–4.28]***
Richest	2.32[1.50–3.58]***	7.51[3.25–17.34]***	1.43[0.67–3.07]	3.98[2.74–5.77]***
**HH has electricity**				
Yes	1	1	1	1
No	1.26[1.00–1.58]*	1.42[1.04–1.93]*	1.10[0.78–1.56]	1.53[1.30–1.79]***
**HH has refrigerator**				
Yes	1	1	1	1
No	1.15[0.92–1.44]	1.19[0.93–1.51]	0.90[0.69–1.19]	1.17[0.99–1.38]
**HH has car/truck**				
Yes	1	1	1	1
No	0.93[0.73–1.20]	1.09[0.75–1.58]	0.70[0.52–1.95]*	0.95[0.79–1.15]
**Type of floor material**				
Cement	1	1	1	1
Any form	1.17[0.93–1.47]	0.61[0.45–0.83]***	1.16[0.91–1.47]	0.81[0.70–0.95]**
Sand/wood	1.01[0.88–1.17]	1.55[1.11–2.16]**	1.29[0.88–1.87]	1.29[1.21–1.00]***
**Number of HH members**	0.95[0.91–0.99]*	0.92[0.87–0.98]**	1.03[0.97–1.09]	0.97[0.94–1.04]

Analysis adjusted for all explanatory variables (child, individual women, and household characteristics). aPR denotes adjusted Prevalence Ratio from Double Selection Least Absolute Shrinkage and Selection Operator Poisson regression analysis. 1 = Reference category used for inferences, HH = Household, and GT. Accra = Greater Accra. P-value Notation

*p-value < 0.05

**p-value ≤0.01 and

***p-value ≤ 0.001.

#### The relative importance of risk factors associated with access to hygienic disposal of stools among women with children under two years in Ghana, GDHS 2003–2014

Generally, the most important risk factors of HDS from dominance analysis included the age of the child, wealth index, regional disparity, and religion. Analysis showed that the increasing age of the child was the second important risk factor from pooled data and in 2008 and 2014 (Table 3). It constituted 22.4% of the overall impact from pooled data (Weighted Standardized Dominance Statistics (WSDS) = 0.224), and separately accounted for 20.6% and 17.8% in 2008 and 2014 respectively. In 2003, the increasing age of the child was the first important risk factor constituting 33.3% of the overall impact (WSDS = 0.333). Intuitively, the wealth index ranked first among risk factors from the pooled data and in 2008, constituting approximately 33%. In 2003 and 2014, the wealth index ranked second and third among risk factors respectively, corresponding to 18.7% and 13.9% respectively (Table 3). Regional disparity ranked third among risk factors from the pooled data, and in 2003 and 2008. It constituted 19.6% from the pooled data and 17.9% and 13.7% in 2003 and 2008 respectively. However, in 2014, regional disparity ranked first among risk factors contributing 47.8% of the overall impact. Religion contributed 17.9% of the impact from pooled data and 14.9% of the impact in 2003 (Table 3).

**Table 3:** Relative importance of factors significantly associated with hygienic disposal of stools among women with children under two years in Ghana, GDHS 2003–2014.

Key predictor	GDHS year of study			Pooled
	2003	2008	2014	
	WSDS	WSDS	WSDS	WSDS
Age of Child in months	0.333^**1**^	0.206^**2**^	0.177^**2**^	0.224^**2**^
Wealth index	0.187^**2**^	0.326^**1**^	0.139^**3**^	0.300^**1**^
Region	0.179^**3**^	0.137^**3**^	0.471^**1**^	0.196^**3**^
Religion	0.149^**4**^	0.044^**7**^		0.179^**4**^
Currently breastfeeding	0.034^**7**^			0.032^**6**^
HH has electricity	0.054^**6**^	0.045^**6**^		0.049^**5**^
Number of HH members	0.063^**5**^	0.028^**8**^		
Watch television		0.115^**4**^		
Educational level		0.095^**5**^		
Wanted last child		0.001^**10**^		
Current marital status		0.001^**11**^		
Type of floor material		0.002^**9**^		0.008^**7**^
Sex of child				0.007^**8**^
Birth order			0.020^**7**^	
Relationship to HH head			0.061^**5**^	
Number of living children			0.012^**8**^	
Currently working			0.072^**4**^	
Multiple birth				0.005^**9**^
HH has car/truck			0.049^**6**^	

HH = Household; WSDS = Weighted Standardized Dominance Statistics; Superscript denotes rankings of the relative importance of factors significantly associated with access to hygienic disposal of stools.

### Propensity score 1:1 density Kernel-based matching results

**Fig 1:**
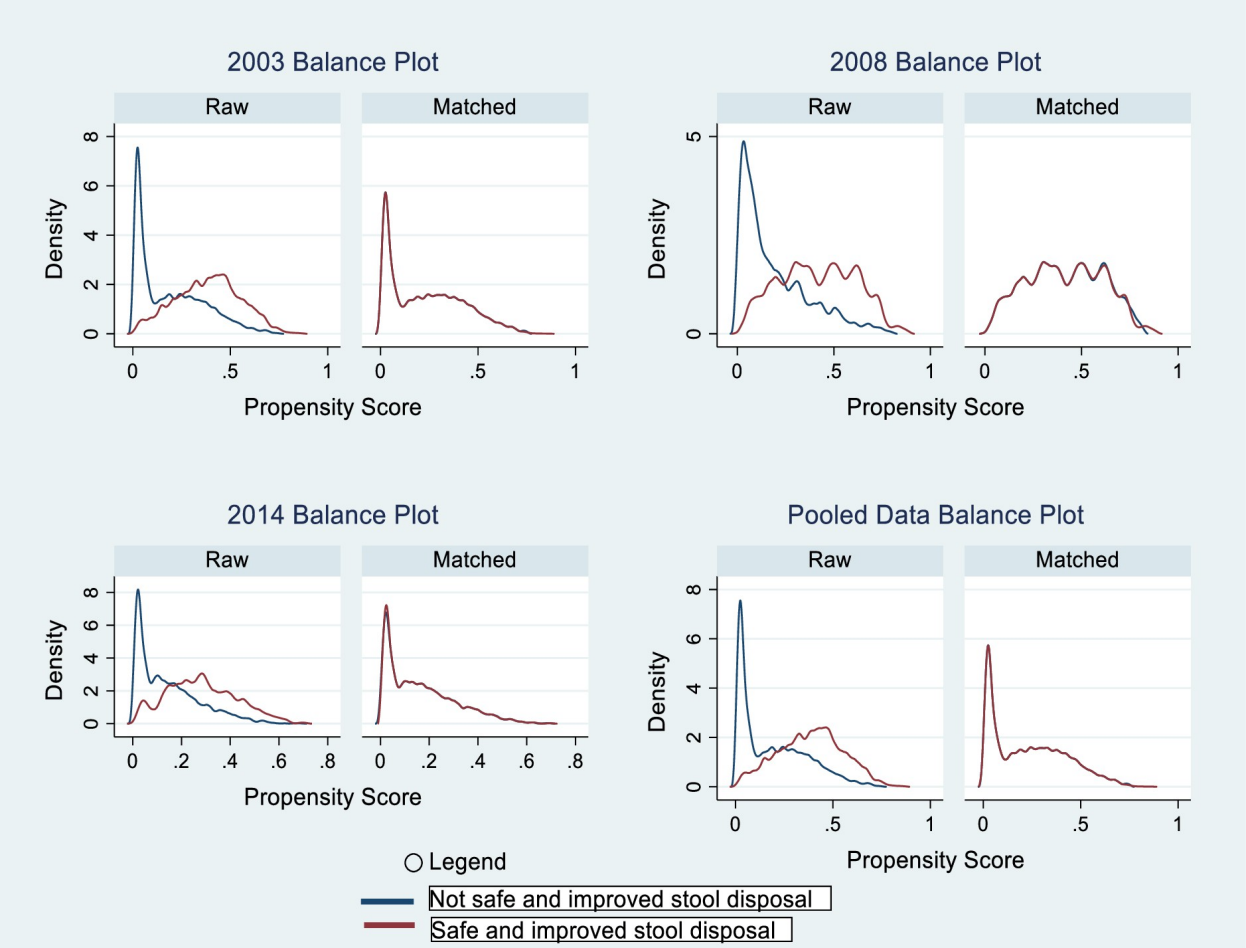
Imbalance and balancing result showing the analytical test of the Kernel-based propensity score matching for covariates associated with access to hygienic disposal of stools among women with children under two years in Ghana, GDHS 2003–2014.

Testing for common support assumption was done before and after Density Kernel-based 1:1 matching procedure. This was done to ascertain how the matching procedure reduced biases in the observed covariates between participants who had access to hygienic disposal of stools and those without. Analysis from Fig 1 clearly showed an overlap for participants who had access to hygienic disposal of stools and those without for each GDHS survey year in addition to the pooled data. However, after the matching procedure, the covariates between the two groups reduced significantly. The results showed that the propensity score with density Kernel-based matching reduced covariate imbalance between the two groups (Fig 1).

### Impact of access to hygienic disposal of stools on the risk of diarrhea diseases

The average diarrheal diseases of children under two years in Ghana whose mothers had access to hygienic disposal of stools reduced over the GDHS year compared to those without access (Table 4). Analysis indicates that in 2003 the average diarrheal diseases of children under two years whose mothers had access to hygienic disposal of stools reduced by 6%, though this was not statistically insignificant (ATE = -0.06, 95%CI = -0.13–0.02). For the subsequent GDHS years (2008 and 2014) in addition with the pooled data, the average reduction rate was observed to be statistically significant [2008 ATE(95%CI) = -0.09(-0.16–0.02), 2014 ATE(95%CI) = -0.05(-0.09–0.01) and Pooled data ATE(95%CI) = -0.05(-0.09–0.02)] (Table 4).

**Table 4:** Treatment effect of access to hygienic disposal of stools on reducing the risk of diarrhea diseases among women with children under two years in Ghana, GDHS 2003–2014.

Hygienic disposal of stools	2003	2008	2014	Pooled
	ATE[95%CI]	ATET[95%CI]	ATET[95%CI]	ATET[95%CI]
Yes vs No	-0.06[-0.13–0.02]	-0.09[-0.16–0.02]**	-0.05[-0.09–0.01]*	-0.05[-0.09–0.02]**

ATET denotes Average Treatment Effect on the Treated (access to hygienic disposal of stools) from Propensity Score Matching (1:1). Impact analysis adjusted for significant factors associated with hygienic disposal of stoolsfor each year of GDHS and the pooled sample. These factors include age of child in months, wealth index, region, religion, currently breastfeeding, household (HH) has electricity, number of HH members, watch television, educational level, wanted last child, current marital status, type of floor material, sex of child, birth order, relationship to HH head, number of living children, currently working, and multiple birth.

## Discussion

### Prevalence of practicing hygienic disposal of stools among women with children under two years in Ghana, GDHS 2003–2014

This analysis investigated the prevalence of HDS and its associated factors and further assessed its impact on diarrheal diseases among children less than two years. From the pooled data analysis, approximately a little more than one-quarter of women with children under two years practice HDS in Ghana. This means about three-quarters of the participants did not practice HDS which calls for urgent attention. In India, a study conducted by Bawankule et al (2017) found approximately four-fifths of participants disposing off stool unsafely [2] which corroborate with the present finding. Bawankule and colleagues involved children under five years while the present study involved children under two years. In North West Ethiopia, a study conducted by Mihrete and friends using similar data found a little more than half (55%) of the participants not disposing off child’s stool safely. Differences between the high non-HDS in our study and finding by Mihrete and friends may be attributed to the definition of the HDS. Our study only involved women with children under two years and HDS was defined as having a safe and improved place for stool disposal while earlier authors considered safe methods of stool disposal only, without including improved latrines. In addition, Sahiledengle (2019) used the fourth round of the Ethiopian DHS in 2016 and found approximately 37% of the participants practicing HDS [16]. Compared with the present analysis, the prevalence rate was approximately 2% less than the values reported for DHS 2003, and 18% less than values reported for GDHS, 2014. The difference could be explained by the age group reported by Sahiledengle (using children under five years) and the DHS year of study (Sahiledengle used 2016 EDHS). In Ghana, Seidu (2021) used the 2014 GDHS and found approximately a quarter of participants practicing safe stool disposal [17]. Compared with the present study, approximately 19% of participants practiced HDS in 2014 GDHS.

### Factors associated with practicing hygienic disposal of stools among women with children under two years in Ghana, GDHS 2003–2014

From the pooled analysis, factors included female sex differential and increasing age of the child, multiple births, currently breastfeeding, regional disparity, wealth index, having access to electricity, and sand/wood type of floor material significantly increased the likelihood of practicing HDS. Consistently, a child’s age, region, wealth index, and HH has electricity were factors significantly associated with HDS throughout the GDHS years. These factors have been associated with stool disposal both in Ghana and elsewhere [2, 7, 17, 23].

The present analysis has bridged the existing knowledge gap on factors associated with HDS by adopting dominance analysis to exhibit the relative importance of the risk factors. The findings indicate that overall, the most important risk factors of HDS were the age of the child, wealth index, and regional disparity. Analysis showed that the wealth index was the first important risk factor from the pooled data during 2003 to 2014 and in 2008. The increasing age of the child was the second important risk factor from pooled data and in 2008 and 2014. In 2003, increasing age of the child was the first important risk factor constituting approximately 33% of the overall impact. The regional disparity was the third important risk factor from the pooled data during 2003 to 2014, in 2003 and 2008. Among the aforementioned indicators, as the age of the child and wealth index increases, the prevalence ratio for practicing HDS increases. Regional disparity showed a strong inequality of practicing HDS among the regions in the north. Residing in the regions of the north was associated with a lower prevalence of practicing HDS. A probable explanation could be the practice of open defecation in parts of the northern regions as documented by WaterAid (2009) as well as relatively low socioeconomic status and availability of hygienic stool disposal facilities in the domestic settings. Certain sociocultural belief systems do influence the use of some public toilet facilities, as demonstrated in the observations of Water Aid. In their survey, people claimed that ‘public toilets are possessed by evil spirits and should be avoided,’ whereas some believed that ‘latrine usage would deprive the consumer of their magical abilities. Others defecate in the open to shield their bodies from the noxious odors and stenches emanating from the toilet/pit latrine, which they do not tolerate even close to their homes [10]. These observations resulted in the adoption of innovative strategies including Community Led Total Sanitation (CLTS) to end open defecation. Despite considerable progress made, open defecation continues in some parts of the country, with all the potential health risks.

### Impact of hygienic disposal of stools on the risk of diarrhea disease

A wide variety of communicable diseases are transmitted and distributed primarily by how human stool is handled [24]. Diseases may be transmitted through direct contact or animal contact with human excreta if they are not contained or disposed off safely using improved latrines [25]. As a result, proper handling of children’s stools are critical in avoiding diarrheal disease transmission. The prevalence of diarrhea disease among women with children under two years from 2003 to 2008 increased significantly, however, the overall growth showed a significant decline. This finding is congruent with a finding by Tetteh and co-authors in Jasikan, a district located in the Oti Region in Ghana [9]. They found a declining rate of diarrheal disease from 2012–2016 among participants. However, the overall decline of 11.4% established in the present analysis was over 10-fold higher than the estimate in the Jasikan study. In addition, the declining rate of diarrheal diseases from the present analysis contradicts findings by [26] in the Central Region of Ghana; they found increased reported cases of diarrheal diseases from 2008–2012.

This analysis affirms the notion that improving water, sanitation, and hygiene practices are critical for the reduction of diarrheal diseases in young children [27]. The present analysis, has, in addition demonstrated a significant impact of HDS in reducing the risk of diarrhea disease among young children. We found that the prevalence of diarrheal diseases among children under two years in Ghana whose mothers practiced HDS reduced significantly in all periods of the GDHS as well as in the pooled data. Evidence regarding the association between unsafe stool disposal and the high burden of infectious diseases exists [24, 28, 29]. Congruent with the findings of the present analysis, some authors found that the odds of experiencing childhood diarrhea disease was significantly higher than that of children whose stools were disposed off safely [2, 23, 30]. These observations are key and useful for advocacy at the local government level for the improvement of sanitary facilities at the household level. The analysis has provided empirical evidence of the contribution of HDS to the reduction of diarrheal diseases, and that the significant decline in the percentage of mothers practicing HDS has negative consequences on the health and wellbeing of their children.

It is thus not surprising that, since 1999, Ghana has had a National Environmental Sanitation Policy with principal components of sanitary disposal of wastes, including solid wastes, liquid wastes, human excreta, industrial wastes, health care, and other hazardous wastes [6, 31]. The policy document suggested that due to the critical importance of preventing contamination caused by inappropriate human excreta disposal, bye-laws requiring households to build domestic toilets would be strictly enforced [6, 31]; the bane to improvement in hygiene at the household level, has been non-or-limited enforcement of the bye-laws. Evidence provided by the analysis of national survey data provides strong justification for the strict enforcement of the local assemble bye-laws. Public health interventions targeted at the dominant factors identified would be useful in reducing the incidence of diarrheal disease, e.g. toilet training of a child as the child grows older, with the finding that increasing age was associated with HDS. The containment of faeces by ensuring households have improved toilets requires some capital for installation, therefore local government support and contribution from development partners and the private sectors would be key. In the long term, addressing income inequalities and structured national interventions of rural and urban slum development are worthy considerations by national and regional authorities.

**Fig 2:**
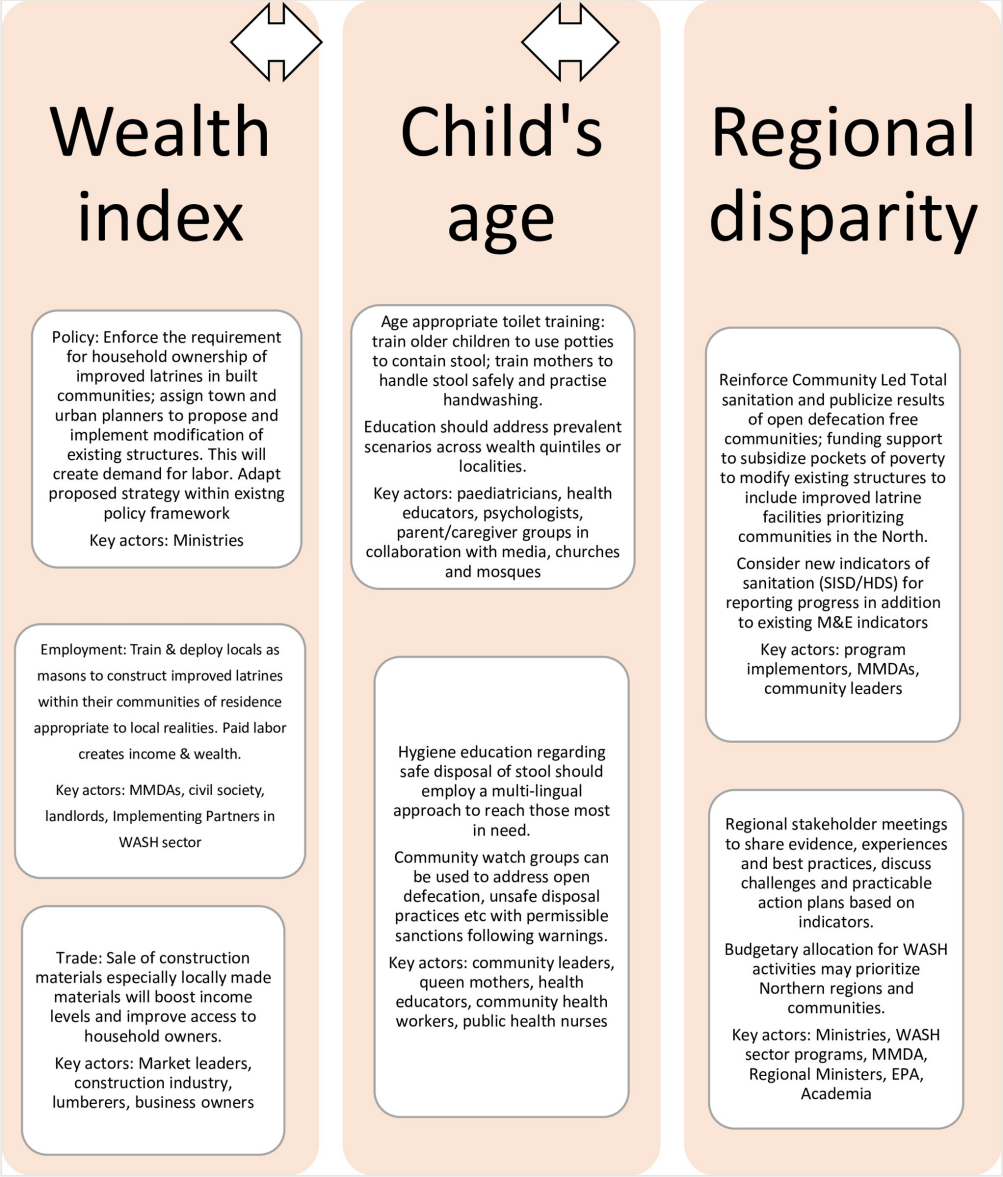
A conceptual framework of a proposed synergy of interventions based on results of the study.

A notable current intervention is the Ghana WASH Project funded by USAID under the auspices of the Ministry of Local Government and Rural Development (MLGRD), which aimed to improve water and sanitation facilities and to increase hygiene education to prevent the spread of diseases including diarrhea by 2025 [25]. Arriving at an evidence-based and practicable synergy of approaches will require dialogue by parent/caregiver groups, community leaders, landlords, Ministry of Health, Ministry of Works and Housing, Ministry of Sanitation and Water Resources, Ministry of Finance, Ministry of Local Government and Rural Development, Ministry of Environment, Science, Technology and Innovation, Ghana Environmental Protection Agency, civil society, and implementing partners in the WASH sector among others. An example of a proposed synergy of interventions is depicted in Fig 2.

## Strengths and limitations

The strength of this study is the use of secondary data which was a nationally stratified survey with appropriate probabilistic sampling methods enabling external validity of the results. The study proposes an enhanced indicator for the exposure (practicing hygienic disposal of stools) and outcome (diarrheal disease prevalence) variables for estimates at the national level. In addition, a notable strength of the current study is the adoption of robust statistical analysis and the matching procedure employed to assess the impact of HDS on diarrhea disease among children under two years. The reduction of imbalance in covariates between treated (participants who practice HDS) and control (who did not) increases the likelihood of estimating the causal effect of HDS in reducing diarrhea episodes. The matching procedure adopted equates or balances the distribution of covariates in the treated and control groups to serve as an experimental study.

A limitation of this study is that the impact of other key measures including handwashing with soap, good personal and food hygiene, health education about how infections spread, and rotavirus vaccination which also contribute to the reduction of diarrheal diseases was unaccounted for in the present analysis [14]. The GDHS did not collect information on the aforementioned measures. In addition, HDS, diarrheal diseases reporting, and disposal of stools were by subjective reporting from GDHS and may be influenced by recall bias. Authors believed, however, that the period for recall was the two weeks preceding the survey, and that recall bias is likely to be minimal.

### Conclusion

Nearly three-quarters of the women with children under two years did not practice HDS from the pooled data analysis. The pattern of HDS showed a significant decreasing rate with health and social policy implications. Interventions can rely on the relative importance of significant factors associated with HDS to reverse the declining rate. In addition, this analysis provides empirical evidence of the positive impact of safe and improved children’s stools practices on childhood diarrhea in a low-income setting to support public engagement in essential environmental sanitation services. The declining rate of HDS raises questions about the effectiveness of the implementation of the 1999 Environmental Sanitation Policy in Ghana. Achievement of the WASH agenda in this low-income setting requires a synergy of interventions and collaborations of actors (government, private, and development partners) to reverse the declining rate of hygienic disposal of stools and risk of diarrheal diseases in young children at the household level.

## Supporting information

S1 Fig Conceptual framework defining the analytical approach for assessing the impact of having access to safe and improved stool disposal among women with children under two years in Ghana, GDHS 2003–2014. (DOCX)

S1 Table Child, individual and household characteristics among women with children under two years in Ghana, GDHS 2003–2014. (DOCX)

S2 Table Forecasting for hygienic disposal of stool and diarrhea infection for the next GDHS. (DOCX)
