# Correction: Hygienic disposal of stools and risk of diarrheal episodes among children aged under two years: Evidence from the Ghana Demographic Health Survey, 2003–2014

**DOI:** 10.1371/journal.pone.0315111

**Published:** 2024-12-03

**Authors:** John Tetteh, Isaac Adomako, Emilia Asuquo Udofia, Elom Yarney, Henry Quansah, Anita Ohenewa Yawson, Akye Essuman, Alfred Edwin Yawson

In the Results subsection of Abstract, there is an error in the seventh sentence. The correct sentence is: The average prevalence of diarrheal diseases among children of women who practiced HDS significantly reduced over the period of the GDHS compared to those whose mothers did not practice HDS [2008 ATE(95%CI) = -0.09(-0.16, -0.02), 2014 ATE(95%CI) = -0.05(-0.09, –0.01) and Pooled data ATE(95%CI) = -0.05(-0.09, –0.02)].

In the Impact of access to hygienic disposal of stools on the risk of diarrhea diseases subsection of Results, there is an error in the second and third sentences. The correct sentences are: Analysis indicates that in 2003 the average diarrheal diseases of children under two years whose mothers had access to hygienic disposal of stools reduced by 6%, though this was not statistically insignificant (ATE = -0.06, 95%CI = -0.13, 0.02). For the subsequent GDHS years (2008 and 2014) in addition with the pooled data, the average reduction rate was observed to be statistically significant [2008 ATE(95%CI) = -0.09(-0.16, -0.02), 2014 ATE(95%CI) = -0.05(-0.09, -0.01) and Pooled data ATE(95%CI) = -0.05(-0.09, -0.02)] (Table 4).

There are errors in the values of 95% Confidence Interval in Table 4. Please see the correct Table 4 here.

**Table 4:** Treatment effect of access to hygienic disposal of stools on reducing the risk of diarrhea diseases among women with children under two years in Ghana, GDHS 2003–2014.

Hygienic disposal of stools	2003	2008	2014	Pooled
	ATE[95%CI]	ATET[95%CI]	ATET[95%CI]	ATET[95%CI]
Yes vs No	-0.06[-0.13, 0.02]	-0.09[-0.16, -0.02]**	-0.05[-0.09, -0.01]*	-0.05[-0.09, -0.02]**

ATET denotes Average Treatment Effect on the Treated (access to hygienic disposal of stools) from Propensity Score Matching (1:1). Impact analysis adjusted for significant factors associated with hygienic disposal of stoolsfor each year of GDHS and the pooled sample. These factors include age of child in months, wealth index, region, religion, currently breastfeeding, household (HH) has electricity, number of HH members, watch television, educational level, wanted last child, current marital status, type of floor material, sex of child, birth order, relationship to HH head, number of living children, currently working, and multiple birth. P-value Notaion: *p-value<0.05, **p-value≤ 0.01
