# Supplementary material for: Hygienic disposal of stools and risk of diarrheal episodes among children aged under two years: Evidence from the Ghana Demographic Health Survey, 2003–2014
Source: PLoS One. 2022 Apr 7;17(4):e0266681. doi: 10.1371/journal.pone.0266681 (PMC8989342; doi:10.1371/journal.pone.0266681)
Supplement: S1 Fig — (DOCX) [file pone.0266681.s001.docx]

S1 Figure 1: Conceptual framework defining the analytical approach for assessing the impact of having access to safe and improved stool disposal among women with children under two years in Ghana, GDHS 2003-2014
