# Supplementary material for: Hygienic disposal of stools and risk of diarrheal episodes among children aged under two years: Evidence from the Ghana Demographic Health Survey, 2003–2014
Source: PLoS One. 2022 Apr 7;17(4):e0266681. doi: 10.1371/journal.pone.0266681 (PMC8989342; doi:10.1371/journal.pone.0266681)
Supplement: S1 Table — (DOCX) [file pone.0266681.s002.docx]

S1 Table 1: Child, individual and household characteristics among women with children under two years in Ghana, GDHS 2003-2014

| **Variable** | **GDHS year of study** | | | **Total** | **Rao-Scott test** |
| --- | --- | --- | --- | --- | --- |
|  | **2003** | **2008** | **2014** |  |  |
|  | Weighted % | Weighted % | Weighted % | Weighted % |  |
| **Sex of child** |  |  |  |  | 0.625 |
| Male | 51.1 | 49.9 | 51.7 | 51.1 |  |
| Female | 48.9 | 50.1 | 48.3 | 48.9 |  |
| **Age of Child in months** |  |  |  |  | 0.692 |
| ≤6 | 28.6 | 31.5 | 30.1 | 30.0 |  |
| 7-11 | 21.9 | 21.9 | 21.7 | 21.8 |  |
| 12-23 | 49.4 | 46.6 | 48.2 | 48.2 |  |
| Mean±SD |  |  |  |  |  |
| **Birth order number** |  |  |  |  | 0.463 |
| 1st | 21.6 | 22.0 | 22.3 | 22.0 |  |
| 2nd | 20.8 | 23.6 | 21.9 | 22.0 |  |
| 3rd | 16.6 | 17.7 | 18.4 | 17.7 |  |
| 4th+ | 41.1 | 36.7 | 37.5 | 38.3 |  |
| **Multiple birth** |  |  |  |  | 0.980 |
| No | 98.1 | 98.1 | 98.0 | 98.0 |  |
| Yes | 1.9 | 1.9 | 2.0 | 2.0 |  |
| **Age of mother** |  |  |  |  | 0.039 |
| Mean±SD | 28.99±6.99 | 28.53±6.63 | 29.32±6.67 | 29.03±6.76 |  |
| **Highest educational level** |  |  |  |  |  |
| No education | 40.8 | 31.0 | 26.9 | 31.9 | <0.001 |
| Primary | 22.1 | 24.1 | 19.0 | 21.1 |  |
| Secondary | 36.1 | 42.0 | 49.4 | 43.8 |  |
| Higher | 1.0 | 2.9 | 4.7 | 3.2 |  |
| **Religion** |  |  |  |  | <0.001 |
| Christian | 89.0 | 72.0 | 74.6 | 78.1 |  |
| Islam | 4.2 | 18.4 | 18.1 | 14.2 |  |
| No religion | 6.8 | 9.6 | 7.2 | 7.7 |  |
| **Relationship to HH** |  |  |  |  | 0.267 |
| Head | 15.8 | 18.4 | 15.4 | 16.3 |  |
| Wife | 66.1 | 64.7 | 68.8 | 67.0 |  |
| Other | 18.1 | 16.8 | 15.8 | 16.7 |  |
| **Currently pregnant** |  |  |  |  | 0.167 |
| No | 96.7 | 97.3 | 95.8 | 96.4 |  |
| Yes | 3.3 | 2.7 | 4.2 | 3.6 |  |
| **Number of living children** |  |  |  |  | 0.022 |
| Mean±SD | 3.18±2.014 | 2.94±1.77 | 3.03±1.85 | 3.05±1.87 |  |
| **Wanted last child** |  |  |  |  | <0.001 |
| Wanted then | 54.0 | 56.3 | 65.4 | 59.9 |  |
| Wanted later | 26.5 | 26.8 | 26.5 | 26.5 |  |
| Wanted no more | 19.6 | 16.9 | 8.1 | 13.5 |  |
| **Currently breastfeeding** |  |  |  |  | 0.004 |
| No | 7.9 | 11.6 | 12.0 | 10.7 |  |
| Yes | 92.1 | 88.4 | 88.0 | 89.3 |  |
| **Current marital status** |  |  |  |  | <0.001 |
| Never married | 3.4 | 6.8 | 8.7 | 6.7 |  |
| Married | 90.9 | 89.6 | 87.3 | 88.9 |  |
| DSW | 5.7 | 3.7 | 4.0 | 4.4 |  |
| **Currently working** |  |  |  |  | <0.001 |
| No | 16.5 | 13.1 | 25.3 | 19.8 |  |
| Yes | 83.4 | 86.4 | 74.7 | 80.0 |  |
| Missing | 0.1 | 0.5 | 0.0 | 0.2 |  |
| **Reads newspaper/magazine** |  |  |  |  | 0.274 |
| No | 90.3 | 88.8 | 88.1 | 88.9 |  |
| Yes | 9.7 | 11.2 | 11.9 | 11.1 |  |
| **Listen to radio** |  |  |  |  | 0.472 |
| No | 16.9 | 19.0 | 17.9 | 17.9 |  |
| Yes | 83.1 | 80.9 | 82.1 | 82.1 |  |
| Missing | 0.0 | 0.1 | 0.0 | 0.0 |  |
| **Watch television** |  |  |  |  | <0.001 |
| No | 54.2 | 46.1 | 30.1 | 40.9 |  |
| Yes | 45.8 | 53.7 | 69.9 | 59.1 |  |
| Missing | 0.1 | 0.2 | 0.0 | 0.1 |  |
| **Sex of Household** |  |  |  |  | 0.205 |
| Male | 74.6 | 72.8 | 76.6 | 75.1 |  |
| Female | 25.4 | 27.2 | 23.4 | 24.9 |  |
| **Age of household head** |  |  |  |  | 0.331 |
| ≤29 | 20.6 | 23.7 | 20.3 | 21.2 |  |
| 30-39 | 36.6 | 37.7 | 39.9 | 38.4 |  |
| 40-49 | 20.9 | 21.3 | 21.6 | 21.3 |  |
| 50+ | 21.8 | 17.3 | 18.2 | 19.0 |  |
| Missing | 0.1 | 0.0 | 0.0 | 0.0 |  |
| **Region** |  |  |  |  | 0.400 |
| Western | 8.9 | 9.5 | 9.5 | 9.4 |  |
| Central | 8.3 | 10.7 | 11.6 | 10.4 |  |
| GT. Accra | 10.8 | 11.3 | 14.7 | 12.8 |  |
| Volta | 9.2 | 9.3 | 7.8 | 8.5 |  |
| Eastern | 10.0 | 9.1 | 9.2 | 9.4 |  |
| Ashanti | 17.2 | 17.8 | 17.3 | 17.4 |  |
| Brong Ahafo | 11.1 | 9.1 | 9.3 | 9.8 |  |
| Northern | 14.9 | 13.5 | 13.5 | 14.2 |  |
| Upper east | 3.5 | 4.2 | 4.2 | 4.3 |  |
| Upper west | 6.2 | 2.9 | 2.9 | 3.8 |  |
| **Place of residence** |  |  |  |  | 0.006 |
| Urban | 33.6 | 38.6 | 44.6 | 40.0 |  |
| Rural | 66.4 | 61.4 | 55.4 | 60.0 |  |
| **Wealth index** |  |  |  |  | 0.432 |
| Poorest | 27.1 | 24.0 | 22.8 | 24.3 |  |
| Poorer | 22.4 | 22.2 | 21.1 | 21.7 |  |
| Middle | 19.1 | 19.0 | 19.1 | 19.1 |  |
| Richer | 16.5 | 20.4 | 19.7 | 19.0 |  |
| Richest | 14.9 | 14.4 | 17.3 | 15.9 |  |
| **HH has electricity** |  |  |  |  | <0.001 |
| No | 64.8 | 50.3 | 30.7 | 45.2 |  |
| Yes | 35.0 | 49.7 | 69.3 | 54.7 |  |
| Missing | 0.2 | 0.0 | 0.0 | 0.1 |  |
| **HH has refrigerator** |  |  |  |  | <0.001 |
| No | 86.2 | 78.3 | 69.8 | 76.5 |  |
| Yes | 13.6 | 21.7 | 30.2 | 23.4 |  |
| Missing | 0.2 | 0.0 | 0.0 | 0.1 |  |
| **HH has car/truck** |  |  |  |  | 0.004 |
| No | 95.6 | 95.1 | 91.6 | 93.6 |  |
| Yes | 4.4 | 4.6 | 8.4 | 6.4 |  |
| Missing | 0.0 | 0.3 | 0.0 | 0.1 |  |
| **Type of floor material** |  |  |  |  | <0.001 |
| Cement | 65.0 | 61.2 | 63.2 | 63.2 |  |
| Any form of carpet | 8.0 | 18.9 | 26.5 | 19.4 |  |
| Sand/wood | 26.0 | 19.1 | 7.1 | 15.4 |  |
| Missing | 1.1 | 0.8 | 3.2 | 2.0 |  |
| **Number of HH members** |  |  |  |  | 0.004 |
| Mean±SD | 6.02±0.09 | 5.63±0.11 | 5.59±0.12 | 5.72±2.70 |  |

NOTE: HH denote Household and GT. Accra represent Greater Accra. SD represent Standard deviation.
