# Supplementary material for: Hygienic disposal of stools and risk of diarrheal episodes among children aged under two years: Evidence from the Ghana Demographic Health Survey, 2003–2014
Source: PLoS One. 2022 Apr 7;17(4):e0266681. doi: 10.1371/journal.pone.0266681 (PMC8989342; doi:10.1371/journal.pone.0266681)
Supplement: S2 Table — (DOCX) [file pone.0266681.s003.docx]

S1 Table 2: Forecasting for hygienic disposal of stool and diarrhea infection for the next GDHS

| Next GDHS | Estimate |
| --- | --- |
| HDS | 8.1 |
| Diarrhea infection | 13.5 |

**NOTE**: Abbreviation; HDS= Hygienic Disposal of Stool; GDHS= Ghana Demographic Survey
